# Cex1 is a component of the COPI intracellular trafficking machinery

**DOI:** 10.1242/bio.058528

**Published:** 2021-03-22

**Authors:** Ludovic Enkler, Bruno Rinaldi, Johan Owen de Craene, Philippe Hammann, Osamu Nureki, Bruno Senger, Sylvie Friant, Hubert D. Becker

**Affiliations:** 1 Génétique Moléculaire et Cellulaire, Université de Strasbourg, CNRS, GMGM UMR7156, F-67000 Strasbourg, France; 2 ‘Architecture et Réactivité de l'ARN’, Université de Strasbourg, CNRS, Institut de Biologie Moléculaire et Cellulaire, F-67000 Strasbourg, France; 3 Department of Biophysics and Biochemistry, Graduate School of Science, The University of Tokyo, Tokyo 113-0032, Japan

**Keywords:** Cex1, COPI coat, Trafficking, SCYL1, Arc1

## Abstract

COPI (coatomer complex I) coated vesicles are involved in Golgi-to-ER and intra-Golgi trafficking pathways, and mediate retrieval of ER resident proteins. Functions and components of the COPI-mediated trafficking pathways, beyond the canonical set of Sec/Arf proteins, are constantly increasing in number and complexity. In mammalian cells, GORAB, SCYL1 and SCYL3 proteins regulate Golgi morphology and protein glycosylation in concert with the COPI machinery. Here, we show that Cex1, homologous to the mammalian SCYL proteins, is a component of the yeast COPI machinery, by interacting with Sec27, Sec28 and Sec33 (Ret1/Cop1) proteins of the COPI coat. Cex1 was initially reported to mediate channeling of aminoacylated tRNA outside of the nucleus. Our data show that Cex1 localizes at membrane compartments, on structures positive for the Sec33 α-COP subunit. Moreover, the Wbp1 protein required for N-glycosylation and interacting via its di-lysine motif with the Sec27 β′-COP subunit is mis-targeted in *cex1*Δ deletion mutant cells. Our data point to the possibility of developing Cex1 yeast-based models to study neurodegenerative disorders linked to pathogenic mutations of its human homologue SCYL1.

## Abstract (precis)

**Summary:** Cex1, the yeast homologue of mammalian SCYL1, interacts with COPI coat components and is recruited to the Golgi to regulate retrograde vesicular trafficking and sorting

## INTRODUCTION

In eukaryotic cells, the final destination of proteins at specific intracellular compartment is ensured by different membrane trafficking pathways. Among them, the coat protein II (COPII) pathway is directing trafficking of proteins or lipids from the endoplasmic reticulum (ER) to the *cis*-Golgi. COPII vesicles budding from the ER is controlled by the small GTPase Sar1 (Barlowe and Schekman, 1993; Nakano and Muramatsu, 1989; Feyder et al., 2015). The retrograde pathway that ensures retrieval of some ER resident components, as well as Golgi trafficking of newly modified proteins is mediated by coatomer complex 1 (COPI) coated vesicles and the small GTPase Arf1 (Letourneur, et al., 1994; Kirchhausen, 2000). The COPI complex is made of α-COP (Sec33/Cop1/Ret1), β- (Sec26) β′- (Sec27), γ- (Sec21), δ- (Ret2), ε- (Sec28) and ζ-COP (Ret3) proteins (Cosson and Letourneur, 1994). The HDEL retrieval signal present in soluble ER resident proteins is recognized by the Erd2 receptor. Membrane proteins have a C-terminal KKXX retrieval motif ensuring their retrograde transport by COPI vesicles via the Rer1 receptor. The COPI coat is conserved from yeast to human. A subset of COPI-coated vesicles subunits (Sec28, Sec27 and Sec33) also assemble in a so-called COPIb complex, to target vacuolar protein sorting from the TGN to the vacuole via the CPY pathway (Gabriely et al., 2007).

Cex1 has been identified as a cytoplasmic component of the tRNA export machinery, based on a yeast triple hybrid assay aimed at identifying protein-RNA interactions (McGuire and Mangroo, 2007). Its implication in tRNA nuclear export was supported by TAP (tandem affinity purification) and pull-down experiments in which Cex1 co-purified with Los1, Gsp1, eEF-1A and the nucleoporin Nup116 (McGuire and Mangroo, 2007). Moreover, cells lacking *CEX1* and *LOS1* are impaired in nuclear tRNA export but cell growth was not affected (McGuire and Mangroo, 2007). In addition, previous studies showed that cells bearing deletions of *LOS1* and *ARC1* (a cytoplasmic tRNA export protein) genes are not viable (Simos et al., 1996). These data suggest that Cex1 and Arc1 have distinct functions in tRNA nuclear export. A recent study narrowed down Cex1 implication in tRNA export by showing that it actually re-exports nuclear aa-tRNAs, being a relay from Los1 to eEF1A on the cytoplasmic side of the nuclear pore complexes (NPC) (McGuire and Mangroo, 2007). These functions were also proposed to be conserved in mammalian cells (Chafe et al., 2011).

Recent attempts to link *CEX1* and *ARC1* failed (Johnstone et al., 2011; Costanzo et al., 2016) at reproducing the previously observed *cex1*Δ *arc1*Δ lethality (McGuire and Mangroo, 2007), casting doubt on the existence of a genetic interaction between *ARC1* and *CEX1*. We thus tested the genetic interaction between *CEX1* and *ARC1*. Our data show that *CEX1* is not synthetic lethal with *ARC1*. To better understand the molecular and cellular function of Cex1, we analyzed the interactome of Cex1 by immunoprecipitation followed by mass spectrometry analyses. Our data reveal that Cex1 associates with the COPI complex subunits Sec27, Sec28 and Sec33, and colocalizes with Sec33. Moreover, Cex1 acts as a regulator of the vesicular trafficking required for retrieval of the Wbp1 ER resident membrane protein. Interestingly, these findings corroborate what is known in mammalian cells, where *SCYL1* regulates the Golgi-to-ER transport both in human and mice via its interaction with COPI coat proteins, the Golgi protein GORAB and by recruiting Arf1 (Burman et al., 2008, 2010; Witkos et al., 2019; Amano et al., 2020).

## RESULTS

### *CEX1* is not genetically linked to *ARC1*

**Fig. 1.**
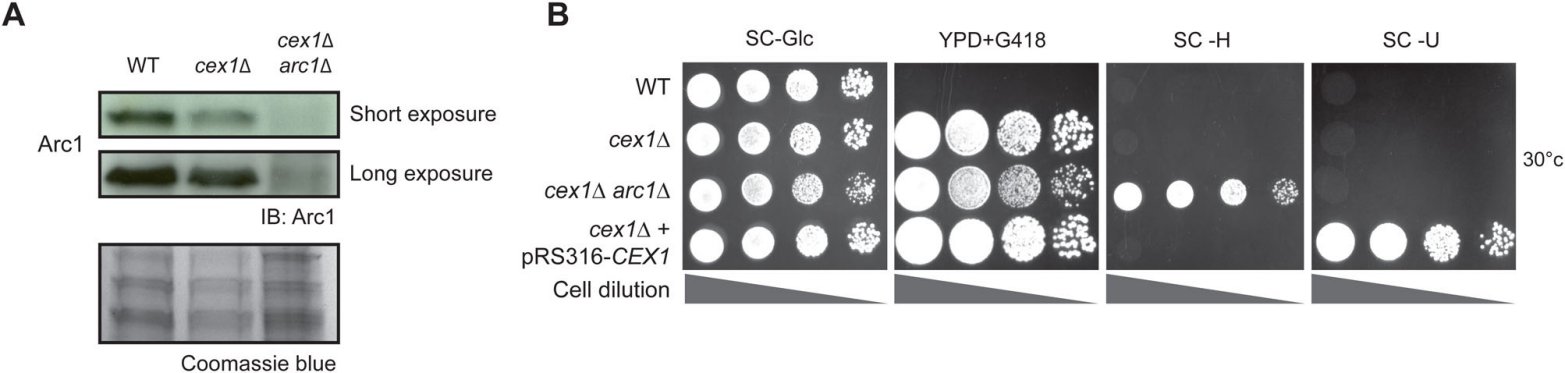
***CEX1* is not genetically linked to *ARC1*.** (A) Immunodetection of Arc1 in a WT (BY4742), *cex1Δ* and *cex1Δ arc1Δ* strains. Coomassie Blue staining was also performed to highlight protein levels in each lane. (B) Drop test of the WT, *cex1Δ*, *cex1Δ arc1Δ* and *cex1Δ*+pRS316-CEX1 strains on rich media in the presence of Geneticin (YPD+G418), and on glucose containing synthetic selective media (SC-Glc, SC-H, SC-U). Growth was performed at 30°C for 2 days.

To analyze the genetic interaction between *ARC1* and *CEX1*, we generated and studied the *cex1*Δ and *cex1*Δ *arc1*Δ deletion strains. Since a previous study showed that the *cex1*Δ *arc1*Δ double deletion was lethal (McGuire and Mangroo, 2007), we first generated a *cex1*Δ strain expressing *CEX1* under its own promoter from a centromeric pRS316 plasmid. Next, *ARC1* was deleted by homologous recombination and absence of *ARC1* was assessed by immunodetection (Fig. 1A). To test for genetic interactions, we grew the *arc1*Δ *cex1*Δ cells bearing the pRS316-CEX1 plasmid on 5-FOA plates to induce plasmid loss. After 3 days of incubation at 30°C, several isolated colonies were observed on the 5-FOA plates suggesting that in our genetic background, *ARC1* and *CEX1* are not synthetically lethal. To further confirm these results, a phenotypic analysis of the different strains was done on different selective media (Fig. 1B).

Since Arc1 was previously associated to the diauxic shift from fermentation to respiration metabolisms (Frechin et al., 2009, 2014), we also tested growth on respiratory medium (SC-Gly) and at different temperatures. Deletion of *CEX1* alone did not impair growth in any tested conditions, whereas the *cex1*Δ *arc1*Δ strain displayed reduced growth on SC-Gly (Fig. S1A), this respiratory defect being due to *ARC1* deletion since the *arc1*Δ cells showed a similar growth delay (Fig. S1A). These experiments also show that *CEX1* is not required for the selection of the carbon source (fermentation versus respiration) during growth of *Saccharomyces* *cerevisiae*. Compared to the wild-type (WT) cells, the *cex1*Δ *arc1*Δ strain did not display any growth defect at 25–30°C on SC-Gly showing that Cex1 is not required for the Los1/Arc1-related pathway of tRNA nuclear export during respiration (Fig. S1A). Further comparative fluorescence microscopy analyses of WT versus *cex1*Δ cells did not detect any obvious morphological defect in the mitochondrial network induced by *CEX1* deletion (Fig. S1B).

### Cex1 interacts with COPI vesicles coat proteins

**Fig. 2.**
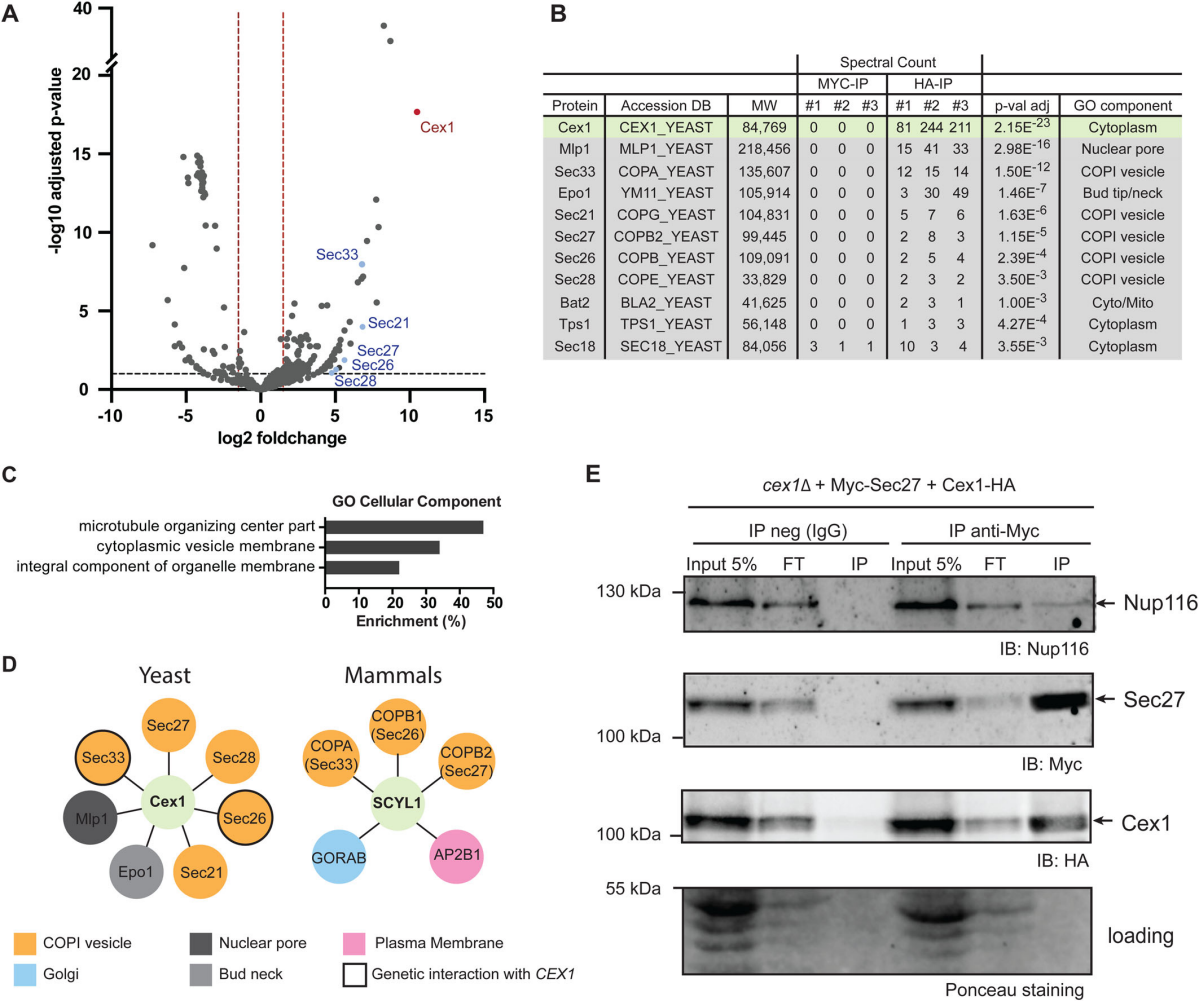
**Cex1 interacts with members of COPI-coat vesicles.** (A) Volcano plot derived from data obtained by IP using Cex1-HA as bait. Three independent IPs were done and proteins were incubated with HA-beads or MYC-beads as control. Hits with at least two peptides, a log2fold over 1.5 and with an adjusted *P*-value below 1% were kept as positive. The graph shows the Cex1 bait (colored in red), together with five components of the COPI coat that co-purified with the bait (colored in blue). (B) Table summarizing Cex1 interactants involved in trafficking. The number of spectral counts detected in each of the three replicates for each protein is shown. MW, molecular weight in Dalton; DB, yeast database. (C) GO term ‘Cellular compartment’ enrichment generated form data obtained in A. Total enrichment was calculated by normalizing positive targets from the IP to the entire proteome of *S. cerevisiae* (obtained from the *Saccharomyces* Genome Database). (D) Comparison of yeast (Cex1) and mammalian (SCYL1) interactome. Proteins from COPI vesicles are shown in orange, Golgi proteins in blue, proteins from plasma membrane are in pink and proteins from the nuclear pore or from the bud neck are in grey and light grey, respectively. Genes genetically linked to *CEX1* based on the work of Costanzo and colleagues (2006) are circled. (E) Interaction between Sec27-Myc and Cex1-HA. The β′-COP Sec27-Myc immunoprecipitation was performed on *cex1*Δ cells expressing Cex1-HA grown in fermentation conditions. Nup116 (nuclear pore complex), Sec27 and Cex1 proteins were immunodetected (indicated by an arrow), and a Ponceau staining of the blot was done as loading control. The negative control IP was done by using IgG instead of anti-Myc antibodies. The input (total protein extract, prior IP), flow-through (FT) and IP fractions were analyzed.

To better understand the cellular role of Cex1, we performed immunoprecipitation (IP) followed by mass-spectrometry analyses using a *cex1*Δ strain with a chromosomally-encoded HA-tagged version of Cex1, allowing expression of Cex1-HA under the dependence of its own promoter (Tables 1 and 2). Cells were grown and lysed in the presence of mild detergent to retrieve potential membrane-associated complexes. Based on mass-spectrometry spectral counts, the most abundant proteins belong to the COPI vesicle family, namely: Sec21, Sec26, Sec27, Sec28 and Sec33 (Gabriely et al., 2007) (Fig. 2A,B, also see Table S1). Cellular component GO term analysis of Cex1 interactants revealed that proteins participating in microtubule organizing center part, cytoplasmic vesicle's membranes and integral component of organelle's membrane were enriched in our interactomics experiments, respectively, at 47%, 34% and 22% (Fig. 2C). In these experiments, Epo1, involved in septin-ER tethering, Mlp1, a protein from the nuclear envelope and two cytoplasmic proteins (Bat2 and Tps1), were also detected.

**Table 1.**
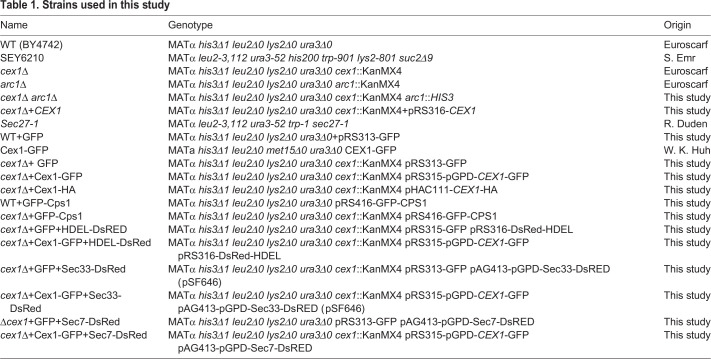
**Strains used in this study**

**Table 2.**
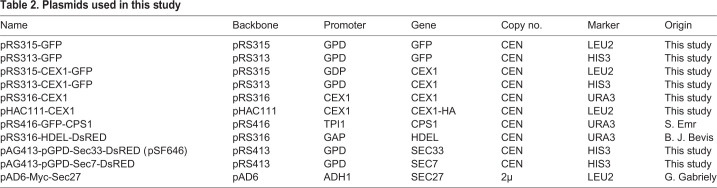
**Plasmids used in this study**

To further assess the connection between Cex1 and vesicular trafficking, we looked for evidences of such interactions in the literature. A recent genetic interaction screen done in *S. cerevisiae* showed that 16 genes are genetically linked to *CEX1* and among them eight encode Golgi and/or COPI vesicle coat proteins (Usaj et al., 2017). Moreover, *SEC33* and *SEC26* are also genetically linked to *CEX1* (Usaj et al., 2017) (circled names, Fig. 2D). The mammalian homologue of Cex1, SCYL1, also interacts with human COPI components, COPA (Sec33), COPB1 (Sec26) and COPB2 (Sec27) (Hamlin et al., 2014) (Fig. 2D). Based on Saccharomyces Genome Database (SGD) and BioGRID3.4, *CEX1* has 15 known physical interactants, and among them four were retrieved in our interactomic analysis. Using the same databases, we retrieved 90 genetic interactants, a third (37) being linked to the Golgi apparatus and involved in intracellular trafficking. Almost half (15) of these 37 genetic interactants were also retrieved as physical partners in our interactomic study using Cex1-HA as bait (Fig. S2A).

A co-IP assay was done to test the interaction between the Sec27 subunit of COPI vesicles (Fig. 2E), and Cex1 or the nucleoporin Nup116 required for nucleocytoplasmic transport, as Cex1 was previously described as a component of the tRNA nuclear export machinery (McGuire and Mangroo, 2007). Protein lysates of *cex1*Δ+pHAC-CEX1+pAD6-MYC-SEC27 were incubated with Sepharose beads coated with anti-Myc or with non-specific immunoglobulin (IP-neg IgG) control, and the immunoprecipitates were analyzed by western blot with anti-HA, anti-Nup116 or anti-MYC antibodies. The results show that Sec27 interacts with Cex1. We also observe a weak interaction with Nup116 that could be linked to the role of Cex1 in nuclear aa-tRNAs export at the cytoplasmic side of the nuclear pore complexes (NPC) (McGuire and Mangroo, 2007). Next, interaction was tested between Cex1 and clathrin, the major coat protein involved in plasma membrane endocytosis and Golgi trafficking, or the ATPase Kar2/BiP an ER protein chaperone (Fig. S2B). The Kar2/BiP chaperone has a KDEL sequence at its C-terminus ensuring its recognition by the KDEL receptor (Lewis and Pelham, 1992) for its transport back to the ER by the COPI vesicles (Orci, et al., 1997). Lysates from *cex1*Δ+Cex1-HA were IP with anti-HA or without antibodies (IP-neg) as control. Kar2 and the clathrin heavy chain Chc1 endogenous proteins were detected using specific polyclonal antibodies. Despite strong affinity purification of Cex1-HA, we could not detect interaction with clathrin Chc1, showing that interactions with COPI components are specific. Moreover, we also did not detect interaction with Kar2, a soluble protein that is retrieved to the ER via COPI mediated trafficking. Taken together, our results show that Cex1 interacts with several subunits of the COPI coat complex.

### Intracellular localization of Cex1

**Fig. 3.**
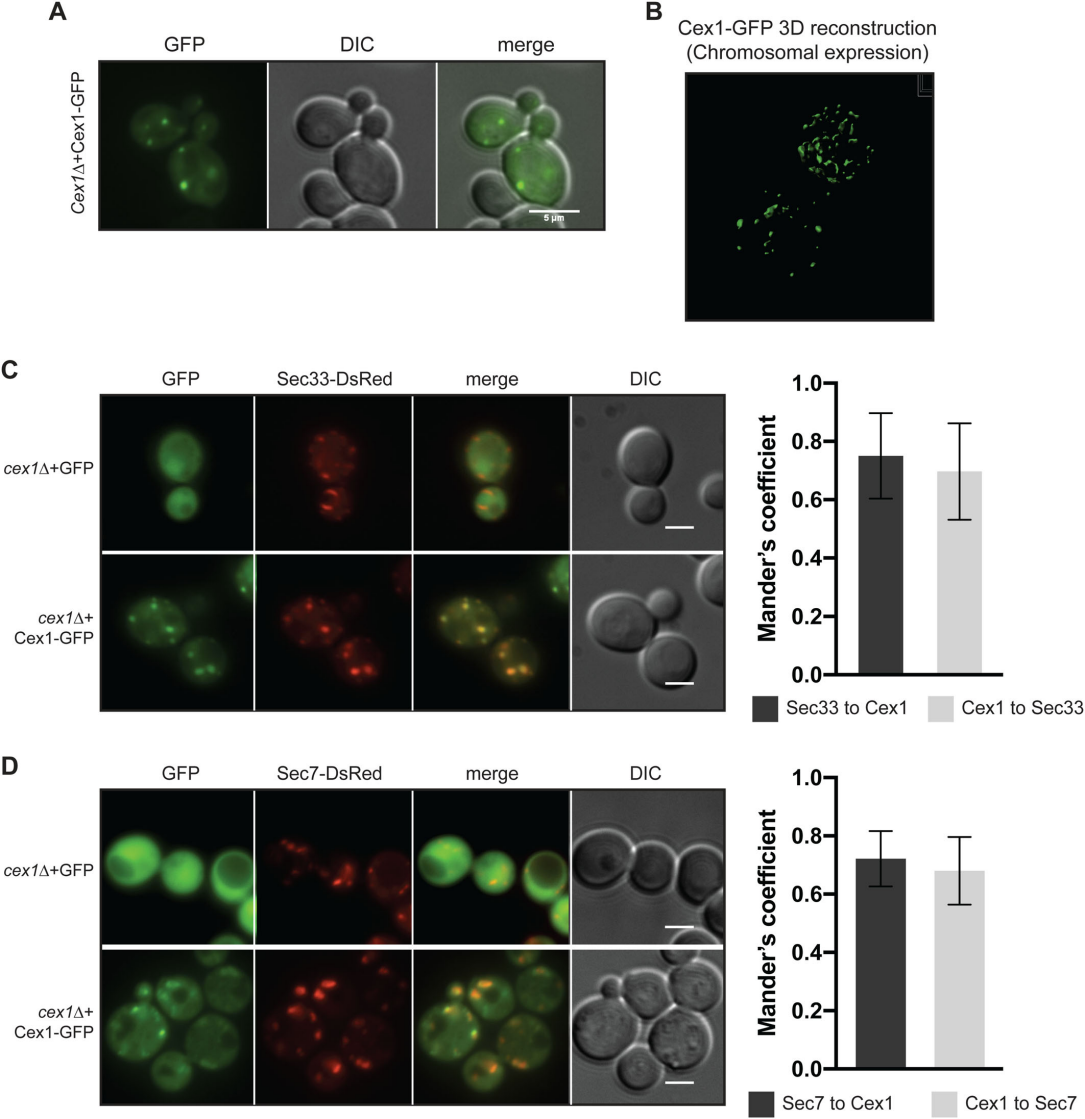
**Cex1-GFP colocalizes with the α-COP Sec33-DsRED.** (A) Epifluorescence microscopy of Cex1-GFP expressed in a *cex1*Δ strain. (B) 3D reconstitution of the Cex1-GFP construct when expressed at its genomic locus. (C) The Sec33 (Cop1/Ret1) COPI component was tagged with DsRED and its intracellular localization analyzed upon Cex1-GFP expression. *cex1*Δ cells expressing GFP alone were used as control. The colocalization of both markers were measured by Mander's coefficient (right panel). (D) Sec7-DsRED was used as a *trans*-Golgi marker together with Cex1-GFP. Their colocalization was measured by Mander's coefficient (right panel). *cex1*Δ cells expressing GFP alone were used as control. Scale bars: 5 µm. Representative micrographs from analyses done on different independent clones are shown.

To determine the intracellular localization of Cex1, we constructed a C-terminally tagged Cex1-GFP fusion protein, since based on the tridimensional 3D-structure of Cex1, linking GFP after the disordered C-terminal domain of Cex1 should not impair its cellular functions (Nozawa et al., 2013). Next, we analyzed Cex1 cellular localization using the Cex1-GFP fusion protein expressed in the *cex1*Δ strain (see Tables 1 and 2, Fig. 3A). In these conditions Cex1-GFP was mainly localized in punctuated structures. We sought to determine if the endogenous localization of Cex1 was similar to that of the overexpressed version. To do so, we used a strain in which *CEX1* was chromosomally GFP-tagged and confirmed the presence of Cex1-GFP in punctate by confocal microscopy followed by Z-stack acquisition and 3D reconstruction (Fig. 3B). However the very low GFP signal, most probably due to the low abundance of Cex1 molecules per cell (Ghaemmaghami et al., 2003), rendered this analysis difficult. Nevertheless, the Cex1-GFP signal showed that Cex1 forms punctate structures in the cytoplasm of yeast, suggesting that Cex1 could be localized to membrane structures.

We then studied the subcellular localization of Cex1 by using the Cex1-GFP fusion protein expressed in the *cex1*Δ strain. DAPI staining of living yeast cells expressing Cex1-GFP or GFP (used as a negative control) showed that the Cex1-GFP positive fluorescent dots do not colocalize with nuclei nor with mitochondria DNA (Fig. S3A). Based on our Co-IP experiments showing interactions between Cex1 and COPI components, we then determined whether Cex1 co-localizes with the COPI subunit or a TGN marker. Microscopic observations show that Cex1 co-localizes with Ret1/Cop1 Sec33-DsRed punctuated structures present in the cells (Fig. 3C) and with the TGN marker Sec7 (Fig. 3D). We observed that Cex1 did not co-localize with the vacuolar membrane stained by internalization of the fluorescent lipid dye FM4-64 (Fig. S3B) suggesting that Cex1 interacts with COPI proteins and colocalizes with Sec33 α-COP and Sec7.

### Cex1 is associated to membrane fractions and regulates Golgi-to-ER trafficking

**Fig. 4.**
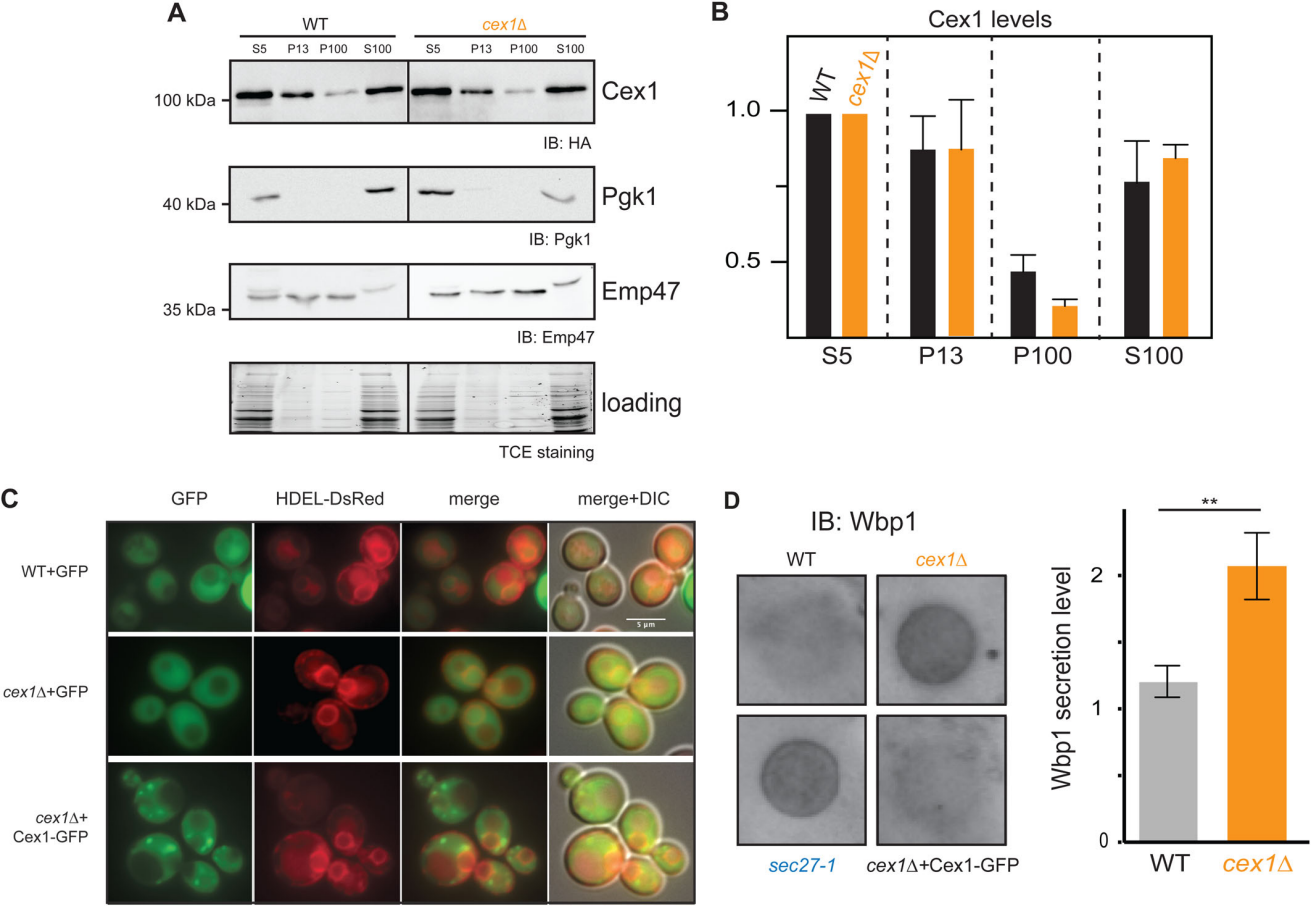
**Cex1 is associated to membrane fractions, and its deletion leads to defective trafficking of Wbp1, an ER protein having a di-lysine COPI sorting signal.** (A) Subcellular fractionation of WT (SEY6210) and *cex1*Δ mutant strain, expressing the Cex1-HA fusion protein. Immunodetection of Cex1-HA in each fraction was done using anti-HA antibodies. Presence of cytosolic proteins in the different fraction was assessed by detecting the soluble Pgk1 protein, and ER as well as ER-Golgi vesicles resident protein by the integral membrane protein Emp47. Protein loading was controlled by TCE staining. (B) Cex1 protein levels detected in the P13, P100 and S100 fractions in A were measured using the total lysate (S5) as standard for each strain. Three independent replicates (*n*=3) were used. Standard deviation is shown. Statistical analyses using the *t*-test was done; **P<0.01; ***P<0.001. (C) Intracellular localization of the HDEL-DsRED reporter localized at the ER via COPI trafficking was assessed by fluorescence microscopy. Scale bar: 5 µm. Representative micrographs are shown from analyses done on independent clones. (D) Trafficking of Wbp1, an effector of N-glycosylation bearing a KK COPI retrieval motif, was assessed by a dot-blot assay on WT, *sec27-1*, *cex1*Δ and *cex1Δ*+Cex1-GFP cells grown in fermentation conditions (SC medium, at 25°C). Statistical analysis from data obtained from three independent clones was done using the *t*-test; **P=0.01.

To determine whether this Cex1-GFP fluorescent punctuate staining corresponded to membrane association, we performed subcellular fractionation and followed Cex1-HA distribution. The differential centrifugation yielded four fractions, the total S5 (supernatant 500× ***g***), the P13 fraction (13,000× ***g*** pellet) recovering ER, mitochondria, nucleus, vacuoles and plasma membrane, the P100 fraction (100,000× ***g*** pellet) recovering Golgi and endosomes and the S100 fraction (supernatant 100,000× ***g***) recovering cytosol and vesicles. Cex1 distribution was followed by expressing *CEX1* under its own promoter and fused to HA tag in WT (SEY6210) yeast cells and *cex1*Δ. In both strains, Cex1-HA was mainly detected in the P13 membrane and in the S100 fractions, and much less in the P100 fractions, showing no differences in localization (Fig. 4A,B).

The COPI vesicles are required for Golgi to ER retrograde trafficking of soluble proteins having a HDEL retrieval motif. In both WT and *cex1*Δ mutant cells, the HDEL-DsRed was localized at the ER (Fig. 4C), showing that Cex1 is not required for COPI-dependent HDEL retrieval. We also did not detect an interaction between Cex1 and Kar2/BiP, an ER chaperone having a KDEL sequence (Fig. S2B). Moreover, the Cex1-GFP signal did not colocalize with HDEL-DsRed (Fig. 4C). A subset of transmembrane ER-resident proteins bearing di-lysine motifs are also transported via COPI vesicles from the Golgi apparatus back to the ER (Cosson and Letourneur, 1994). Among them, the Wbp1 membrane protein is required for N-glycosylation in the ER and interacts via its classical di-lysine motif KKTN, with the N-terminal WD40 propeller domains of the Sec27 β′-COP subunit (Eugster et al., 2000). In mammalian cells, SCYL1 homologous to yeast Cex1 regulates protein glycosylation in concert with the COPI machinery (Witkos et al., 2019). Impairment of membrane trafficking at the ER-Golgi level leads to several defects among them mis-targeting via the secretory machinery to the plasma membrane. To test if Cex1 is involved in ER retrieval of Wbp1, we analyzed its mis-targeting to the plasma membrane via a colony dot blot assay (Fig. 4D). The latter shows that the *sec27-1* COPI mutant strain and the *cex1*Δ strain exhibited Wbp1 mis-targeting via the secretory pathway in contrast to the WT cells and to the *cex1*Δ strain expressing Cex1-GFP (Fig. 4D). However, the sorting of the carboxypeptidase S (Cps1) via the vacuolar protein sorting pathway (VPS) was not impaired since Cps1 was properly localized to the vacuolar lumen in both WT and *cex1*Δ cells (Fig. S4A). These data suggest a role for Cex1 in the Golgi-to-ER pathway but not the Golgi-to-vacuole pathway.

## DISCUSSION

Based on previous reports, we sought to identify the genetic link between *ARC1* and *CEX1* in the nuclear export of aa-tRNA (McGuire and Mangroo, 2007, 2012; Nozawa et al., 2013). To do so we generated a strain deprived of both *CEX1* and *ARC1* using the same genetic background as previously described (McGuire and Mangroo, 2007). However, we did not observe synthetic lethality between these two genes in our tested conditions. Besides our report, other studies also revealed the absence of a genetic link between *ARC1* and *CEX1* (Costanzo et al., 2016; Johnstone et al., 2011). Of note, a recent large-scale genetic interaction network consisting in nearly 1 million genetic interactions was constructed in *S. cerevisiae* (Costanzo et al., 2016). This high-throughput study, using deletion and temperature-sensitive mutant for essential genes, did also not report a genetic link between *ARC1* and *CEX1.* This finding challenges the biological role attributed to Cex1 with regards to that of Arc1 in the export of aa-tRNA in *S. cerevisiae*; therefore to better understand the cellular function of Cex1, we analyzed the interaction network and localization of Cex1 in the yeast *S. cerevisiae*.

Here we show that Cex1 is a cytoplasmic protein that interacts with components of the COPI vesicular coat and localizes at α-COP (Sec33/Cop1/Ret1) and TGN positive punctate structures (Figs 2,3; Table S1). Among the five COPI subunits identified by our interactomic study, Sec33 was the strongest interactant of Cex1 (Fig. 2A,B), while the other subunits (Sec21, 26, 27 and 28) showed a lower but still significant enrichment. Since COPI subunits assemble into a stable complex, this would imply that Cex1 interacts primarily with Sec33 and that the other subunits are likely enriched through Sec33 direct interactions, but not to Cex1. Consistent with our present findings, the orthologues of Cex1 in mammals (SCYL1, 2 and 3), are also constituents of vesicular complexes and regulate Golgi-to-ER transport and Golgi morphology both in Human and mice cells via their interaction with COPI coat proteins in part mediated by PRMT1 arginine methylation (Burman et al., 2008, 2010; Amano et al., 2020). A similar role has also been suggested through an ORFeome analysis of GFP-tagged proteins for the orthologue Ppk32 in the fission yeast *Schizosaccharomyces pombe* (Matsuyama et al., 2006), later confirmed by brefeldin A sensitivity of *ppk32*Δ mutant cells (Kowalczyk and Petersen, 2016).

The discrepancy between our findings that Cex1 is related to COPI coat in yeast, and the current literature stating that it is involved in the export of aa-tRNA from the nucleus, can be reconciled if Cex1 has a dual role in trafficking or if the latter is operated through COPI vesicles but this has never been observed and seems unlikely since COPI vesicles emanates from the Golgi compartment. However, in our co-immunoprecipitation experiments, we observed a weak binding between Sec27 and Nup116, a nuclear pore component (Fig. 2E). Past studies on Cex1 might have missed COPI-related proteins due to technical issues. Here, we solubilized membranes with a rather high amount of detergent (1% NP40) as compared to classical buffers used to recover cytosolic proteins (see Material and Methods). This led to the solubilization of membrane-bound complexes and allowed detection of a large number of proteins involved in intracellular traffic.

Based on our data, we envision that Cex1 could participate in the binding of some COPI proteins at the Golgi membrane, to ensure sorting and recycling of some di-lysine motif containing ER resident membrane proteins. In mammalian cells, SCYL1 is recruited by the Golgi protein GORAB and this is crucial for the subsequent recruitment of Arf1-GTP and the COPI coat complex (Witkos et al., 2019). A recent observation shows that pseudokinase domains, despite lacking their catalytic activity, still retained the ability to bind and/or hydrolyze ATP (Murphy et al., 2014). This ATP-binding property is sought to regulate a proportion of pseudokinase-dependent signaling, either through modulation of catalytic activity, or by conformational transition (Byrne et al., 2017; Hammarén et al., 2015). Cex1, which contains such a pseudo kinase domain (Nozawa et al., 2013), could then act as an ATP-exchange factor, as a substrate trap or as a mobile platform to promote protein-protein interactions at the Golgi. Regardless of its genuine molecular function, Cex1 regulates the retrograde vesicular trafficking and sorting at the Golgi level of some ER resident proteins. This is supported by several lines of evidence: (1) Cex1 interacts with three components of the COPI coat (Fig. 2), (2) it co-localizes with Sec33 α-COP protein (Fig. 3), (3) and cells lacking *CEX1* exhibit mis-targeting of the ER resident Wbp1 protein (Fig. 4).

SCYL1, SCYL2 and SCYL3 are members of the SCY1-like (SCYL) family of mammalian pseudokinase proteins, and several studies suggested that they somehow participate to the regulation of protein trafficking along the secretory pathway (Conner and Schmid, 2005; Düwel and Ungewickell, 2006; Borner et al., 2007; Burman et al., 2008, 2010; Hamlin et al., 2014). Even if SCYL1 was not detected in a recent proteomic analysis of COPI-coated vesicles (Gilchrist et al., 2006), it has to be noted that peripheral membrane proteins associating with organelles are not always identified by MS analyses (Blondeau et al., 2004). Members of the SCYL family harbor variants of the dilysine motif KKxx-COO- responsible for COPI binding (Burman et al., 2008). It has also been shown that internal KK motifs present in the C-terminal helical region are involved in the binding of cytoplasmic proteins to β’COP protein (Sullivan et al., 2000). Interestingly Cex1 possess a WDTNW motif at its very C-ter end that resembles the unique Wx_n(1–6)_[WF] motif of the δ-COP subunit of coatomer and to that of SCYL3 (Suckling et al., 2015 ; Kuliyev et al., 2018), further rationalizing the observation that Cex1 is interacting with COPI vesicles.

Development of neurodegenerative diseases is sometimes associated with dysfunction of the intracellular trafficking apparatus (Ferrucci et al., 2011; Majcher et al., 2015; Dahm and Macchi, 2007) and in human as well as in mouse, mutations in the *SCYL1, SCYL2* and *SCYL3* genes have been linked to amyotrophic lateral sclerosis (ALS) and other neuronal dysfunction and activity (Pelletier et al., 2012; Schmidt et al., 2015; Kuliyev et al., 2018). In human mutations in *SCYL1* were associated to peripheral neuropathy, cerebellar atrophy, and ataxia (Schmidt et al., 2015). *SCYL1*-deficient mice are called muscle deficient (*mdf*), and are defined by an early onset of progressive motor neuron disorder (Schmidt et al., 2007). The *Scyl1^mdf/mdf^* mice display cytosolic accumulation of TDP-43 and Ubiquilin 2 and have symptoms similar to those observed in ALS patients (Pelletier et al., 2012). The fact that a protein regulating COPI trafficking and Golgi morphogenesis is somehow involved in ALS both in Human and mice opens a new road to better understand the development of this disease. Unfortunately, there are no studies aiming to dissect the link between SCYL1 intracellular trafficking and ALS. It would be interesting to study the role of SCYL1 or its yeast homologue in the case of ALS in the light of recent findings describing their role in protein sorting at the Golgi apparatus.

## MATERIALS AND METHODS

### Media and growth conditions

The following rich media were used for the growth of yeast strains: 1% (w/v) yeast extract, 1% (w/v) peptone, 40 mg/l adenine, 2% (w/v) glucose (YPD). We also used synthetic complete medium (SC) composed of 0.17% (w/v) yeast nitrogen base without amino acids and ammonium sulfate, 0.5% (w/v) ammonium sulfate, 2% (w/v) glucose (Glc) or glycerol (Gly) and 0.8% (w/v) of a mixture of amino acids and bases from MP Biomedicals. The solid media contained 2% (w/v) agar. WT (BY4742) and *cex1*Δ mutant strains were grown at 30°C with rotational shaking to mid-log (OD_600nm_ 0.7) or log phase (OD_600nm_ 1.5) depending on the experiments. Prior to subcellular fractionation, *sec27-1* temperature-sensitive strain was grown at 25°C to mid-log phase, and then transferred at 37°C for 3 h.

### Drop tests and Wbp1 colony-blot assay

Drop tests were done using 10 ml cultures grown to log phase. Cells were then spun down, diluted in water to a final OD_600nm_ 0.5 and further diluted to the tenth four times. 7 µl of each dilution was spotted onto agarose plates and incubated for at least 2 days at 25, 30 or 37°C.

For Wbp1 colony blot assay, cells were spotted onto SC agarose plates similarly to a drop test. Once dry, plates were covered with Nitrocellulose membrane and incubated for 3 days at 25°C. Nitrocellulose membranes were then rinsed once with water to remove the excess of cells, and without any lysis treatment, membrane were blocked with 5% (w/v) milk in TBS-Tween buffer, and presence of the Wbp1 at the plasma membrane was assessed by western blot using a rabbit anti-Wbp1 antibody (1:5000; kind gift of Riezman Howard, University of Geneva), and HRP-conjugated anti-rabbit antibodies (1:10,000; GE Lifesciences).

### Yeast transformation

50 ml of yeast cells were grown in SC-Glc to mid-log phase at 30°C. Cells were spun down and washed in 1 volume of deionized water (5 min at 3000*× **g*** at room temperature). The pellet was then resuspended in 5 ml of lithium acetate mix (1× TE; 100 mM lithium acetate), spun down (5 min at 3000*× **g*** at room temperature) and the pellet was resuspended in 0.5 ml of lithium acetate mix and transferred to an Eppendorf tube. After 1 h of shaking (orbital, 150 rpm, 30°C), 200 µl of cells were mixed with 10 µl of 10 mg/ml boiled sheared salmon sperm DNA and 1–5 µg of DNA (PCR product or plasmid). The mix was incubated for 30 min on an orbital shaker (150 rpm, 30°C), 1 ml of PEG3500 was added and the mix was again incubated for 30 min. Following this, cells were incubated 10 min at 42°C, spun down (5 min at 3000*× **g*** at room temperature), pellet was resuspended in 200 µl of TE buffer (100 mM Tris-HCl pH 7.5; 10 mM EDTA pH 8) and cells were plated onto selective media and incubated at 25 or 30°C depending on the strain.

### *ARC1* deletion in the *cex1*Δ strain

The *cex1*Δ strain (see Table 1) was used to obtain the *cex1*Δ *arc1*Δ. A *HIS3* cassette containing *S. cerevisiae HIS3* gene with its promoter (312 bp) and terminator (201 bp) was generated by PCR. Using the ARC1-HIS3 Fwd and ARC1-HIS3 Rev primers (see Table 3), 50 bp of homology domain from *ARC1* localized at the ATG and STOP codons were appended. The *HIS3* cassette was amplified using 1 µl of *S. cerevisiae* genomic DNA (50 ng/µl), 0.3 µM of each primer, 4 µl of Phusion Buffer (10×), 0.2 µl of dNTP (25 mM) and 1 U of Phusion DNA polymerase (Thermo Fisher Scientific) in a 20 µl reaction mix. Reaction was performed 30 s at 98°C, then 10 s at 98°C, 20 s at 60°C and 30 s at 72°C for 30 cycles, and ended by 3 min of reaction at 72°C. After PCR purification using the Wizard^®^ SV Gel and PCR Clean-Up System (Promega), 1 µg of PCR product was then transformed in *cex1*Δ +pRS316-CEX1 strain.

**Table 3.**
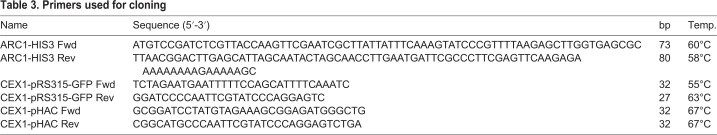
**Primers used for cloning**

### Subcellular fractionation

One hundred ml of cells were grown to mid-log phase in appropriate media and harvested by centrifugation 4 min at 1600*× **g*** at room temperature. Cells were then washed once in 10 ml of cold lysis buffer (20 mM Hepes KOH pH 6.8; 150 mM KoAc; 10 mM MgCl_2_; 250 mM Sorbitol; protease inhibitor cocktail), resuspended in 1 ml of cold lysis buffer and transferred in tubes containing 1/3 volume of glass beads. Cells were broken by mechanical disruption (six cycles at 6 m/s during 30 s) using Fast-Prep (MP Bio), transferred to a new 1.5 ml Eppendorf tube and centrifuged at 300× ***g*** for 5 min at 4°C. Supernatants were transferred to newly tubes and centrifuged at 500× ***g*** for 5 min at 4°C, pellets were resuspended in 200 µl of lysis buffer (P5), aliquots (100 µl) of supernatants were kept for further analysis (S5) and the remaining supernatants were again centrifuged at 13,000× ***g*** for 10 min at 4°C. Pellets (P13) were resuspended in 100 µl of lysis buffer while supernatants were centrifuged for an hour at 100,000× ***g*** at 4°C. Supernatants (S100) were kept for further analysis and pellets (P100) were resuspended in 50 µl of lysis buffer.

### Proteins extraction, immunoprecipitation and western blotting

50 ml of cells grown to log phase were harvested and resuspended in 2 ml of breaking buffer (100 mM Tris-HCl pH 6.8, 150 mM NaCl, 0.5 mM EDTA), supplemented with a cocktail of anti-proteases (cOmplete Mini, EDTA-free, ROCHE) and glass-beads. Cells were broken by mechanical disruption (six cycles at 6 m/s during 30 s) using Fast-Prep (MP Bio). Each aliquot was then incubated 30 min on ice in the presence of 0.5% (v/v) NP40. Protein extracts were obtained after 1 h of centrifugation at 105,000*× **g*** at 4°C.

Prior to immunoprecipitations and western-blots, protein concentrations were determined using Bradford. Co-IP were done on 1 mg of total protein extracts as previously described (Morvan et al., 2015). For western blotting 10–15 µg of proteins were separated by SDS-PAGE on a 10% or 12% gel prior to electroblotting onto Hybond-P membrane (Amersham). Detection was carried out using HRP-conjugated anti-rabbit, anti-mouse or anti-goat antibodies (Bio-Rad), at a concentration of 1:5000. We used ECL-plus reagents (Bio-Rad) according to the manufacturer's instructions. Loading was determined by Coomassie or Ponceau staining of the membrane, or via 2,2,2-trichloroethanol TCE staining (Chopra, et al., 2019). Presence of Pgk1 was probed with a mouse monoclonal anti-Pgk1 antibody (1:5000; PA5-28612-Invitrogen), Cex1-GFP with anti-GFP monoclonal antibodies [1:5000; ab1218, clone (9F9.F9), Abcam], Cex1-HA with a mouse or a rat monoclonal anti-HA antibody [1:10,000, 11867423001, clone (3F10), Roche], Sec27-Myc with anti-Myc mouse monoclonal antibodies [1:5000; 11667149001, clone (9E10), Roche], Arc1 with our polyclonal serum, Chc1 with mouse monoclonal antibodies (1:1000; a kind gift from Sandra K. Lemmon, University of Miami), anti-Nup116 antibodies (1:2000; a kind gift from Susan R. Wente, Vanderbilt University), Kar2/BiP with a polyclonal serum (1:1000; a kind gift from Hugh R. Pelham, MRC Cambridge).

### Fluorescent microscopy analyses and images acquisition

Cells were incubated overnight in appropriate media and living cells in exponential phase of growth were used for microscopy studies. For vacuoles staining, cell cultures were centrifuged 1 min at 1500× ***g*** at room temperature, resuspended in 50 µl of YPD encompassing 4 µl of FM4-64 (200 µM, Life Technologies), and incubated 20 min at rotational shaking at 30°C. Cells were washed in 500 µl of fresh YPD, resuspended in 100 µl of SC-Glc and incubated for another 15 min at 30°C. For vacuolar staining in respiration, cells were treated as described but were incubated 30 min at 30°C, washed in YPGly, resuspended in 100 µl of SC-Gly and incubated for another hour at 30°C. Mitochondria were stained using MitoTracker Red CMXRos (Invitrogen). 1 µl (200 µM stock) was added to 1 ml of culture and cells were incubated 20 min at 30°C with rotational shaking. Cells were then washed with appropriate medium and images were taken. Nuclei were stained with 15 µl of DAPI (10 µg/ml) and incubated 10 min in the dark. Three washes with PBS are needed to avoid non-specific background fluorescence. Observation was performed with a 100×/1.45 oil objective (Carl Zeiss) on a fluorescence Axio Observer D1 microscope (Carl Zeiss) using DAPI, GPF or DsRED filters and DIC optics. Images were captured with a CoolSnap HQ2 photometrix camera (Roper Scientific) and treated by ImageJ (W. S. Rasband, ImageJ, NIH, USA, http://imagej.nih.gov/ij/). Images for 3D reconstruction were taken using a confocal LSM 780 high resolution module Airyscan with a 63×1.4NA plan apochromatic objective (Carl Zeiss) controlled by the Zen Black 2.3 software (Carl Zeiss).

### Cex1 immunoprecipitation

To identify Cex1 partners, three independent clones were grown to log phase and cells were harvested by centrifugation at 4100*× **g*** for 10 min at room temperature. Cells were frozen in liquid nitrogen and lysed with a mortar at 4°C. The cell powder obtained was then resuspended in a lysis buffer (0.33% NP40; 50 mM Tris-HCl pH8; 50 mM NaCl; 1 mM PMSF), supplemented with cOmplete Roche antiproteases, and centrifuged at 12,000× ***g*** for 15 min at 4°C. The supernatant was recovered and 1.35 mg of proteins were mixed with 50 µl of magnetic anti-HA MicroBeads (Myltenyi Biotec) and incubated 30 min on ice. The suspension was then loaded onto µMacs column, washed three times using a modified version of the lysis buffer containing 0.1% (v/v) of NP40 and final elution was done with 50 µl of Laemli buffer boiled at 95°C.

### Mass spectrometry and nano-LC/MS analysis

For nano-LC-MS/MS analysis, the dried extracted peptides were transferred in vials compatible with nano-LC-MS/MS analysis (Ultimate 3000, Dionex and MicroTOF-Q, Bruker). The method consisted in a 60-min gradient at a flow rate of 300 nl/min using a gradient from two solvents: A (0.1% formic acid in water) and B (0.08% formic acid in acetonitrile). The system includes: a 300 µm×5 mm PepMap C18 precolumn (Dionex) in order to pre-concentrate peptides and a 75 µm×150 mm C18 column (Dionex) used for peptide elution. MS and MS/MS data were acquired in a data-dependent mode using Hystar (Bruker) and processed using Mascot software (Matrix Science). Consecutive searches against the NCBI nr database first and then against the *S. cerevisiae* taxonomy were performed for each sample using an intranet version of Mascot 2.0. Peptide modifications allowed during the search were: N-acetyl (protein), carbamidomethylation (C) and oxidation (M). The other parameters were: peptide tolerance=0.4 Da, MS/MS tolerance=0.4 Da, two missed cleavage sites by trypsin allowed. Proteins showing two peptides with a score higher than the query threshold (*P*-value<0.05) were automatically validated with Proteinscape (Bruker). The total number of MS/MS fragmentation spectra was used to quantify each protein from at least three independent biological replicates (Basic Spectral Count). After a column-wise normalization of the data matrix, the spectral count values were submitted to a negative-binomial test using an edgeR GLM regression through R (R v3.2.5). The statistical test was based on the published msmsTests R package available in Bioconductor to process label-free LC-MS/MS data by spectral counts. For each identified protein, an adjusted *P*-value (adjp) corrected by Benjamini–Hochberg was calculated, as well as a protein fold-change (FC). Each protein identified by only one peptide was checked manually using the classical fragmentation rules. Protein bands were manually excised from the gels and transferred into 96-well microtitration plates. Excised gel samples were cut in small pieces and washed three times by incubation in 25 mM NH_4_HCO_3_ for 15 min and then in 50% (v/v) acetonitrile containing 25 mM NH_4_HCO_3_ for 15 min. Samples were then dehydrated with 100% acetonitrile and then reduced with 10 mM DTT during 1 h before being alkylated with 55 mM iodoacetamide for 1 h in the dark. Gel pieces were washed again with the destaining solutions described above. 0.250 µg of modified trypsin (Promega, sequencing grade) in 25 mM NH_4_HCO_3_ were added to the dehydrated gel spots depending on protein amount. After 30 min incubation at room temperature, 20 µl of 25 mM NH_4_HCO_3_ were added on gel pieces before incubation overnight at 37°C. Peptides were then extracted from gel pieces in 20 µl of 50% acetonitrile/5% formic acid. Total enrichment in Fig. 2C was calculated by normalizing positive targets from the IP to the entire proteome of *S. cerevisiae* (obtained from the Saccharomyces Genome Database).

### Statistics

Statistical analyses were done with GraphPad Prism 6 and with a Student’s *t*-test. Every experiment was done in biological triplicates, and standard deviation of the mean (s.d.) is showed on every graph.

## Supplementary Material

Supplementary information
