# Supplementary material for: Cex1 is a component of the COPI intracellular trafficking machinery
Source: Biol Open. 2021 Mar 22;10(3):bio058528. doi: 10.1242/bio.058528 (PMC8015235; doi:10.1242/bio.058528)
Supplement: Supplementary information [file biolopen-10-058528-s1.pdf]

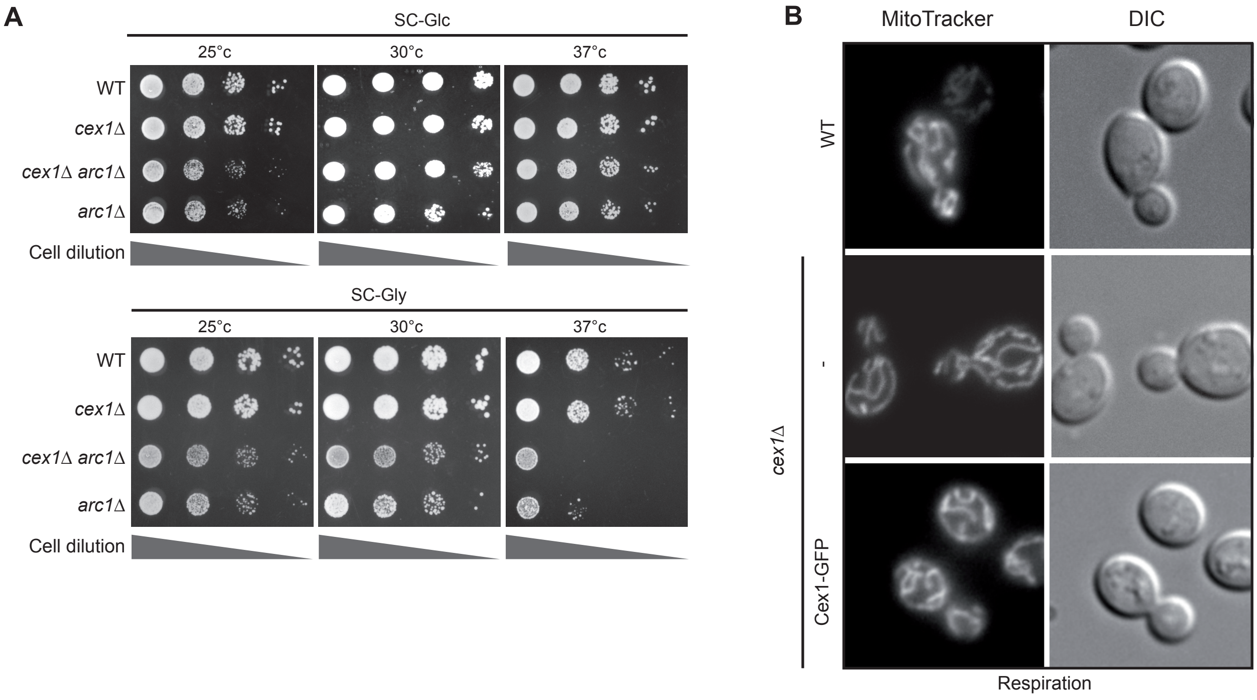

Figure S1. Enkler *et al*

Figure S1 related to Figure 1 and 2: *CEX1* is not genetically linked to *ARF1*, and is involved in intracellular traffic.

A Drop test of the WT, *cex1Δ*, *cex1Δ arc1Δ* and *arc1Δ* strains grown for 2 days in fermentation (SC-Glc) or 3 days in respiration (SC-Gly) at 25, 30 and 37 °C. **B** Mitochondria staining using MitoTracker Red CMXRoS in the wild-type WT strain, *cex1*Δ (-) or the *cex1*Δ expressing Cex1-GFP. Cells were grown in respiration and representative micrographs are shown.

Biology Open • Supplementary information

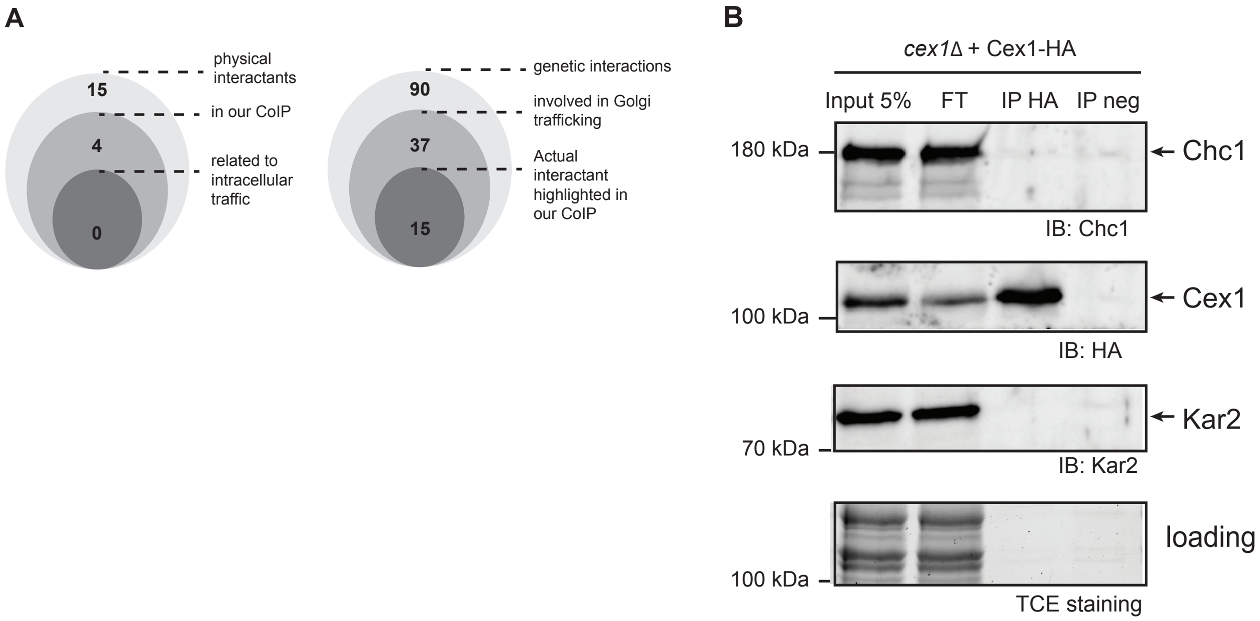

Figure S2 Enkler *et al*

Figure S2 related to Figure 2: Cex1 interacts with members of COPI-coat vesicles

A SGD (Saccharomyces genome database) and BioGrid physical and genetic interactions compared to our interactions data obtained by IP using Cex1 as bait. From the 15 known physical interactants described in the literature, 4 were also retrieved in our data set, but none of these were related to intracellular traffic. Of the 90 known genetic interactions with *CEX1*, 37 are related to genes involved in traffic, and 15 were highlighted as physical interactants in our study. **B** Absence of interaction between Cex1, the clathrin Chc1 and Kar2/Bip. An anti-HA immunoprecipitation was performed on *cex1Δ* cells bearing Cex1-HA grown in fermentation conditions. Chc1 (clathrin heavy chain), Cex1 and Kar2 (a COPI cargo containing a KDEL sequence) proteins were immunodetected (indicated by an arrow), and a TCE staining of the proteins was done as loading control. An arrow indicates the band relative to Cex1-HA, present in the IP-HA but not in the negative control IP (IP neg) performed with beads only.

Biology Open • Supplementary information

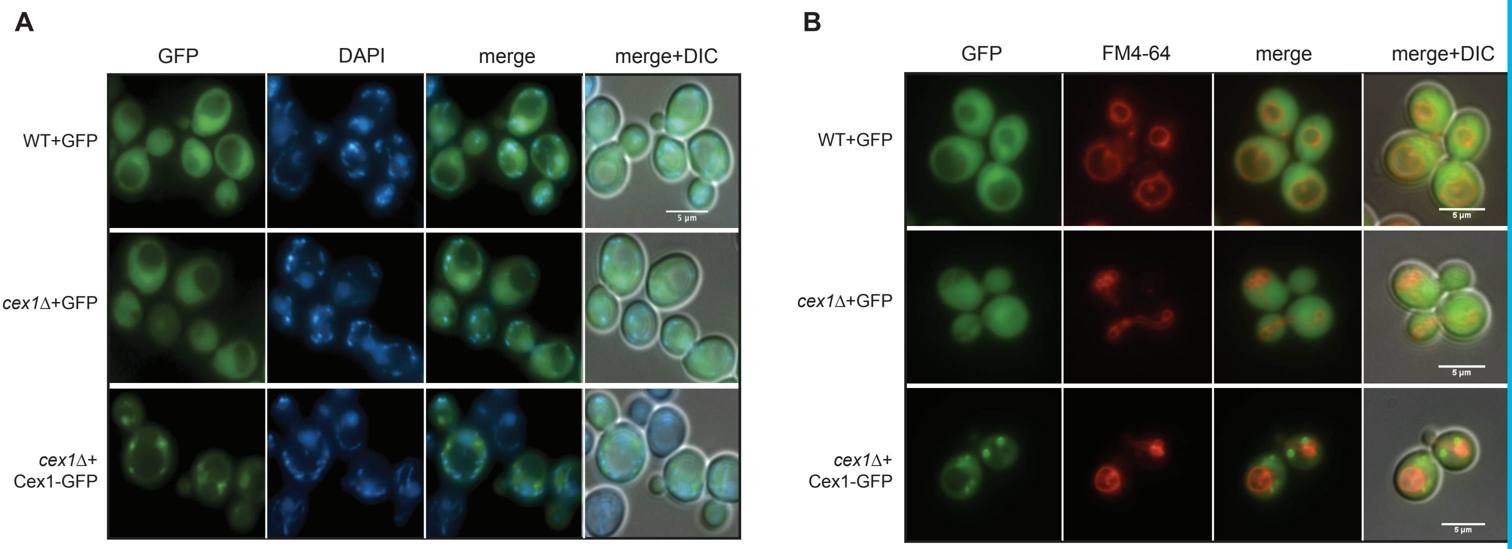

Figure S3. Enkler *et al*

Figure S3 related to Figure 3: Fluorescence microscopy localization of Cex1-GFP and the vacuolar membrane

A Subcellular localization of Cex1-GFP in cells performed after nuclei staining with DAPI. **B** Subcellular localization of Cex1-GFP was performed after staining the vacuolar membranes with the FM4-64 lipid dye. Scale bar: 5 µm. Representative micrographs are shown.

Biology Open • Supplementary information

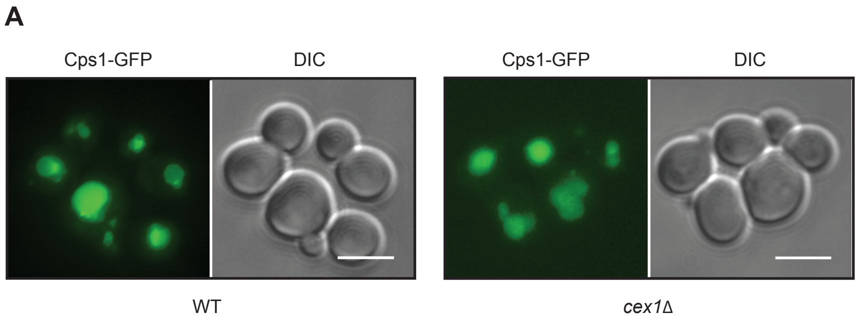

Figure S4. Enkler *et al*

Figure S4 related to Figure 4: *CEX1* disruption does not impact the carboxypeptidase S vacuolar trafficking.

A Localization of the vacuolar carboxypeptidase S (Cps1) fused to the GFP in wild-type WT and *cex1*Δ cells. Scale bar: 5 µm. Representative microg

Table S1

Click here to Download Table S1

Biology Open • Supplementary information
